# Social Isolation and Incident Dementia in the Oldest-Old—A Competing Risk Analysis

**DOI:** 10.3389/fpsyt.2022.834438

**Published:** 2022-06-10

**Authors:** Jessica Grothe, Susanne Röhr, Melanie Luppa, Alexander Pabst, Luca Kleineidam, Kathrin Heser, Angela Fuchs, Michael Pentzek, Anke Oey, Birgitt Wiese, Dagmar Lühmann, Hendrik van den Bussche, Siegfried Weyerer, Jochen Werle, Dagmar Weeg, Horst Bickel, Martin Scherer, Hans-Helmut König, André Hajek, Michael Wagner, Steffi G. Riedel-Heller

**Affiliations:** ^1^Medical Faculty, Institute of Social Medicine, Occupational Health and Public Health (ISAP), University of Leipzig, Leipzig, Germany; ^2^Global Brain Health Institute (GBHI), Trinity College Dublin, Dublin, Ireland; ^3^Department of Neurodegenerative Diseases and Geriatric Psychiatry, University of Bonn, Bonn, Germany; ^4^German Center for Neurodegenerative Diseases (DZNE), Bonn, Germany; ^5^Institute of General Practice (ifam), Medical Faculty, Heinrich Heine University Düsseldorf, Düsseldorf, Germany; ^6^Institute of General Practice, Hannover Medical School, Hannover, Germany; ^7^Department of Primary Medical Care, Center for Psychosocial Medicine, University Medical Center Hamburg-Eppendorf, Hamburg, Germany; ^8^Central Institute of Mental Health, Medical Faculty Mannheim, Heidelberg University, Mannheim, Germany; ^9^Department of Psychiatry, Technical University of Munich, Munich, Germany; ^10^Department of Health Economics and Health Services Research, University Medical Center Hamburg-Eppendorf, Hamburg, Germany

**Keywords:** social isolation, incident dementia, oldest-old, epidemiology, competing risk analysis, longitudinal study

## Abstract

**Purpose:**

Social isolation is considered a risk factor for dementia. However, less is known about social isolation and dementia with respect to competing risk of death, particularly in the oldest-old, who are at highest risk for social isolation, dementia and mortality. Therefore, we aimed to examine these associations in a sample of oldest-old individuals.

**Methods:**

Analyses were based on follow-up (FU) 5–9 of the longitudinal German study AgeCoDe/AgeQualiDe. Social isolation was assessed using the short form of the Lubben Social Network Scale (LSNS-6), with a score ≤ 12 indicating social isolation. Structured interviews were used to identify dementia cases. Competing risk analysis based on the Fine-Gray model was conducted to test the association between social isolation and incident dementia.

**Results:**

Excluding participants with prevalent dementia, *n* = 1,161 individuals were included. Their mean age was 86.6 (*SD* = 3.1) years and 67.0% were female. The prevalence of social isolation was 34.7% at FU 5, 9.7% developed dementia and 36.0% died during a mean FU time of 4.3 (*SD* = 0.4) years. Adjusting for covariates and cumulative mortality risk, social isolation was not significantly associated with incident dementia; neither in the total sample (sHR: 1.07, 95%CI 0.65-1.76, *p* = 0.80), nor if stratified by sex (men: sHR: 0.71, 95%CI 0.28-1.83, *p* = 0.48; women: sHR: 1.39, 95%CI 0.77-2.51, *p* = 0.27).

**Conclusion:**

In contrast to the findings of previous studies, we did not find an association between social isolation and incident dementia in the oldest-old. However, our analysis took into account the competing risk of death and the FU period was rather short. Future studies, especially with longer FU periods and more comprehensive assessment of qualitative social network characteristics (e.g., loneliness and satisfaction with social relationships) may be useful for clarification.

## Introduction

Around 17% of the world population will be 65 years old or older in 2050 (1). The fastest growing group above 65 years of age is the oldest-old, i.e., individuals over 85 years of age (2). Population aging is associated with an increase of age-related disorders, especially dementia (3). Dementia is a neuropsychiatric syndrome that mainly occurs as a result of a degenerative disease of the brain. It is one of the most common and most severe disorders in old age and shortens the life span considerably (4). The number of individuals living with dementia worldwide is constantly increasing (5). In 2015, there were 46.8 million dementia cases (6) and the number is projected to increase to 152 million by 2050 (7). This development will pose major challenges for public health and old age care systems in countries all over the world (8). As there is no effective treatment or cure for dementia yet, increasing costs for health systems and societies at large will emerge (6). Therefore, the importance of dementia risk reduction and prevention is growing.

It is also well known that social isolation increases with age (9). The prevalence of social isolation in community-dwelling older adults ranges from 10 to 43% (10). Social isolation is defined as a low number and frequency of contacts with others (11). It is an objective measure and can be assessed by quantifying an individual's social network. Social isolation is associated with increased mortality (12, 13), an increased risk of developing coronary heart disease and stroke (14).

A number of studies demonstrated a relationship between characteristics and aspects (e.g., social participation, living alone, and less frequent contact) of social network size and cognitive performance as well as incident dementia (15, 16).

Moreover, Holt-Lunstadt et al. (12) showed that individuals with adequate social relationships had a 50% higher probability of survival compared to those with poor or insufficient social relationships. The extent of this effect was comparable to that of smoking cessation and it exceeds many other known risk factors for mortality (e.g., overweight, lack of exercise) (12, 17).

The effect of social isolation on the brain were studied in an experiment with mice. Smith et al. (18) showed that the aging brain can be positively influenced by larger social networks. These findings support the assumption that the social network is associated with brain structure and could thus affect cognitive function and the development of dementia.

In this context, we aimed to longitudinally assess the association between social isolation and incident dementia in oldest-old individuals. To the best of our knowledge, no competing risk analysis has been performed in the oldest-old to investigate the association of social isolation and incident dementia. However, it is important to consider competing events when analyzing survival data in old and oldest-old individuals (19). In particular, mortality is a relevant competing risk in oldest-old individuals when studying the association of health outcomes, including dementia (20).

We study the group of the oldest-old, as they are different from younger older age groups (21). For example, among individuals 90 years of age and older, incidence dementia increases exponentially (2). The oldest-old are at high risk for several risk factors associated with incident dementia (e.g., sensory deficits, frailty, physical disability, malnutrition, and unintentional weight loss) (21). In addition, the risk of social isolation is specifically high in the oldest-old (21).

We assume that those who are not socially isolated are less likely to develop dementia over the course of the study.

We aimed to assess the association between social isolation and incident dementia in the oldest-old longitudinally, taking mortality risk into account.

## Materials and Methods

This work is informed by the STROBE (22) guidelines for reporting observational studies in epidemiology.

### Study Design and Sampling

Analyses were carried out using data of the German study on Aging, Cognition, and Dementia in Primary Care Patients (AgeCoDe), a prospective longitudinal cohort study on mild cognitive impairment (MCI) and dementia, and its extensioncontinuation, the study on Needs, Health Service Use, Costs, and Health-related Quality of Life in a large sample of oldest-old primary care patients (AgeQualiDe). Participants were recruited by 138 general practitioners (GP) in six cities (Bonn, Duesseldorf, Hamburg, Leipzig, Mannheim, Munich) between January 2003 and November 2004. GP patients were eligible for the AgeCoDe/AgeQualiDe-study, if they were aged 75 years or older, dementia-free, and had at least one GP contact within the last year. Patients who only saw their GP at their homes, lived in a nursing home, had a serious illness that was expected to be fatal within 3 months, did not have sufficient knowledge of the German language, were deaf or blind, or were unable to give informed consent, were excluded from participation in the study. The study design has previously been described elsewhere (23).

**Figure 1:**
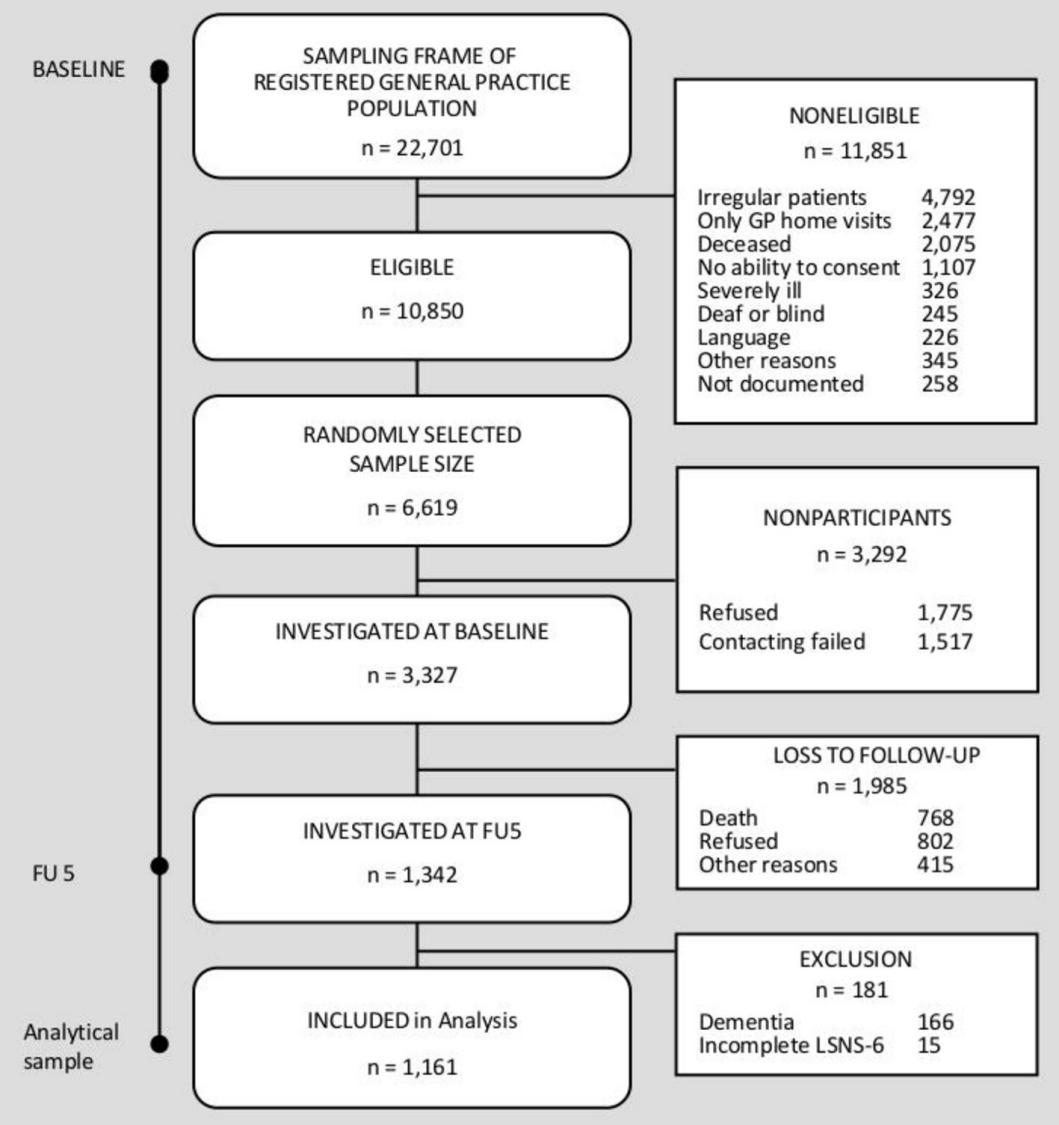
Sample selection flowchart.

Initially, 3,327 individuals constituted the AgeCoDe/AgeQualiDe cohort at baseline. Nine follow-up assessments were scheduled every 1.5 years up to follow-up seven and then every 10 months up to follow-up nine. In this study, waves five to nine were used for the analysis because social network data were only assessed from FU5. At this time, 1,342 individuals were interviewed. For analysis, 181 participants were excluded, because of a diagnosis of dementia at follow-up five (*n* = 166; 91.7%) and missing information on social network, measured by Lubben Social Network Scale (LSNS-6) (*n* = 15; 8.3%). The resulting analytic sample included data from *n* = 1,161 participants. A flowchart of sample selection and attrition is shown in Figure 1.

### Ethics

The ethics committees of all six study centers approved the study. The study was performed in accordance with the ethical standards of the Declaration of Helsinki (24). Patients and/or their proxies provided written informed consent prior to their study participation.

### Instruments

#### Social Isolation

Social isolation was determined by measuring the social network size, using the short form of the Lubben Social Network Scale (LSNS-6). The LSNS correlates with other measures of social integration and thus has good validity (25). It contains questions about the number and frequency of contacts with friends and family as well as social support received by them (25). Each of the six LSNS-6 questions is scored from zero to five. The total score ranges from zero to 30. Higher scores indicate larger social networks. A score below 12 is considered an indicator of social isolation and a score of 12 or higher indicates social integration (25). For this cutoff, the LSNS demonstrates concordant validity for identifying individuals with risk for social isolation (25).

#### Incident Dementia

To identify dementia cases in the AgeCoDe/AgeQualiDe cohort, the Structured Interview for Diagnosis of Dementia of Alzheimer's type, Multi-infarct Dementia and Dementia of other Etiology according to DSM-IV and ICD-10 (SIDAM) interview was used. It contains a neuropsychological test battery (largely comprising the MMSE) and a 14-item scale for the assessment of activities of daily living (SIDAM-ADL-Scale) (26). Dementia was diagnosed in a consensus conference with the interviewer and an experienced geriatrician or geriatric psychiatrist according to the criteria of DSM-IV, which are implemented as a diagnostic algorithm in the SIDAM. Date of follow-up assessment was the point of incident dementia diagnosis.

#### Covariates

Information on several covariates was collected to control for possible confounding effects. Sociodemographic data included age, sex, education (according to the Comparative Analysis of Social Mobility in Industrial Nations (CASMIN) criteria (27)), marital status and living situation. Cognitive function was measured using the Mini-Mental State Examination (MMSE) (28). It consists of 11 questions and activities regarding, e.g., orientation, recall, and visual construction. Higher scores indicate better overall cognitive function. The maximum score is 30. The MMSE was administered as part of the SIDAM (26).

In order to determine the individual's everyday function or functional independence, eight Instrumental Activities of Daily Living (IADL) were collected using the Lawton & Brody IADL scale (29). This scale included, among others, the ability to use a telephone and transportation (car, bus, train), and the ability to manage financial matters. The score ranged from zero to eight. A higher score indicated higher independence.

Cognitive and physical activities were assessed according to Verghese et al. (30) with some small modifications. Activities of the past 4 weeks were collected using an ordinal scale of frequency (four—daily, three—several times per weeks, two—once per weeks, one—less than once per weeks, and zero—never). Physical activities included seven questions, for example on bicycling, walking, swimming, gymnastics, chores/gardening, and a category of other physical activities (e.g., bowling, dancing, bicycling, walking, or golfing). Cognitive activities included eight items, e.g., doing crossword puzzles, memory training/brainteasers, games (card games, board games, or individual games), reading, writing, and playing a musical instrument. For analysis, two sum scores were calculated. One for cognitive and one for physical activities. The score for cognitive activities ranged from zero to 32. The maximum score for physical activities was 28. Higher scores indicated higher activity level.

#### Health Characteristics

Mobility, vision, and hearing impairments were assessed with a self-report question for each domain. Specifically, we asked participants, “Do you have difficulty walking/hearing/seeing?” Responses were recorded using an ordinal scale of severity: (1) no difficulty, (2) some difficulty, (3) significant difficulty, and (4) extreme difficulty or unable to walk/blind/deaf. For analysis, variables were dichotomized (yes/no).

Depressive symptoms were measured using the short version of the Geriatric Depression Scale (GDS) (31). The GDS consists of 15 questions specific to older age, e.g., “have you dropped many of your activities and interests?.” The maximum score is 15 (score > five indicates increased depressive symptomatology; score >10 indicates severe depressive symptomatology).

Information on whether participants had a history of stroke, diabetes mellitus, and hypertension was obtained from standardized questionnaires completed by the participants' general practitioners at each wave of the study.

### Statistical Analyses

Group differences between socially isolated and socially integrated individuals at follow-up five were inspected using Pearson chi-square tests, rank sum tests or Wilcoxon two-sample tests. We used the Fine and Gray (competing risk) regression model to calculate the risk of incident dementia, taking into account the competing event (mortality) over time (19). Fine and Gray's model modifies the Cox proportional hazard model to account for competing risks. A competing risk is understood as an event that hinders the occurrence of the event of interest (32, 33). First, we ran a competing risk analysis without adjustment. In a second step, our competing risk analysis was adjusted for all above named covariates.

In view of the different profiles of risk factors of dementia between women and men previously reported (34), we additionally aimed to conduct analysis stratified by sex.

Results were presented as a sub-distribution hazard ratio (sHR) with a 95% confidence interval (CI). All events except the two of interest (incident dementia and mortality) were censored.

In a sensitivity analysis, we ran all competing risk regression models with social isolation as a time-varying variable.

STATA 16 was used for statistical analysis (35). All analyses employed an α-level for statistical significance of 0.05 (two-tailed).

## Results

### Sample Characteristics

In total, 1,161 dementia-free individuals were included in the analytic sample. Their mean age was 86.57 *(SD* = 3.1) years and *n* = 778 (67.0%) were female. Prevalence of social isolation was 34.7% (*n* = 403) at FU 5, *n* = 113 (9.7%) developed dementia and 418 (36.0%) died during a mean follow-up time of 4.26 (*SD* = 0.35) years. The mean survival time was 3.86 years (*SD* = 1.26). Mortality was higher in socially isolated compared to socially integrated individuals [*n* = 174 (43.2%) vs. *n* = 244 (32.2%); *p* < 0.001].

The average social network size, measured using the LSNS-6 score was 8.07 (*SD* = 2.70) for socially isolated individuals and 17.14 (*SD* = 3.87) for socially integrated participants (*t* = 41.93, *p* < 0.001). Socially isolated individuals were significantly older, less often married, and more often living alone than socially integrated individuals. In addition, they had lower MMSE scores, higher depressive symptoms, less often performed cognitive and physical activities and were more often impaired in vision and mobility. Socially isolated and socially integrated individuals did not differ regarding sex, IADL, history of stroke, history of diabetes mellitus, hypertension and hearing impairment.

Baseline characteristics of the analytical sample are shown in Table 1.

**Table 1:** Sociodemographic and health characteristics of the study sample.

**Variable**	**Total (*n* = 1.161)**	Socially isolated individuals^*^(*n* = 403)	Socially integrated individuals^*^(*n* = 758)	***p*-value**
**Age, M (** ***SD*** **)**	86.6 (3.1)	87.1 (3.1)	86.3 (3.0)	<0.001
**Sex**, ***n*** **(%)**				0.192
Female	778 (67.0)	280 (69.5)	498 (65.7)	
Male	383 (33.0)	123 (30.5)	260 (34.3)	
**Education**, ***n*** **(%)**				0.363
Low	655 (56.4)	232 (57.6)	423 (55.8)	
Middle	357 (30.8)	127 (31.5)	230 (30.3)	
High	149 (12.8)	44 (10.9)	105 (13.9)	
**Marital status**, ***n*** **(%)**				<0.001
Married	346 (29.8)	85 (21.1)	261 (34.4)	
Not married	814 (70.1)	317 (78.7)	497 (65.6)	
Missings	1 (0.1)	1 (0.3)	0 (0.0)	
**Living situation**, ***n*** **(%)**				0.015
Living alone	604 (52.0)	230 (57.1)	374 (49.3)	
Not living alone	557 (48.0)	173 (42.9)	384 (50.7)	
**Cognitive function (MMSE), M (** ***SD*** **)**	27.9 (1.8)	27.6 (2.0)	28.0 (1.7)	<0.001
Missings	7 (0.6)	2 (0.5)	5 (0.7)	
**IADL, M (** ***SD*** **)**	6.5 (1.9)	6.2 (2.09)	6.6 (1.7)	0.052
**Cognitive activities, M (** ***SD*** **)**	12.4 (4.2)	10.8 (3.99)	13.3 (4.1)	<0.001
Missings	20 (1.7)	9 (2.2)	11 (1.5)	
**Physical activities, M (** ***SD*** **)**	6.4 (3.9)	5.4 (3.7)	6.9 (3.9)	<0.001
Missings	25 (2.2)	8 (2.0)	17 (2.2)	
**History of stroke**, ***n*** **(%)**				0.889
Yes	63 (5.4)	22 (5.5)	41 (5.4)	
No	831 (71.6)	283 (70.2)	548 (72.3)	
Missings	267 (23.0)	98 (24.3)	169 (22.3)	
**History of diabetes mellitus**, ***n*** **(%)**				0.278
Yes	252 (21.7)	93 (23.1)	159 (21.0)	
No	635 (54.7)	210 (52.1)	425 (56.1)	
Missings	274 (23.6)	100 (24.8)	174 (23.0)	
**Hypertension**, ***n*** **(%)**				0.425
Yes	761 (65.6)	255 (63.3)	506 (66.8)	
No	135 (11.6)	50 (12.4)	85 (11.2)	
Missings	265 (22.8)	98 (24.3)	167 (22.0)	
**Mobility impairment**, ***n*** **(%)**				<0.001
Yes	460 (39.6)	278 (69.0)	423 (55.8)	
No	701 (60.4)	125 (31.0)	335 (44.2)	
**Hearing impairment**, ***n*** **(%)**				0.694
Yes	567 (48.8)	200 (49.6)	367 (48.4)	
No	594 (51.2)	203 (50.4)	391 (51.6)	
**Vision impairment**, ***n*** **(%)**				0.042
Yes	304 (26.2)	283 (70.2)	184 (24.3)	
No	857 (73.8)	120 (29.8)	574 (75.7)	
**Depressive symptoms (GDS), M (** ***SD*** **)**	2.6 (2.5)	3.5 (2.8)	2.1 (2.2)	<0.001
Missings	16 (1.4)	10 (2.5)	6 (0.8)	
**Mortality**, ***n*** **(%)**	418 (36.0)	174 (43.2)	244 (32.2)	<0.001
**Incident dementia**, ***n*** **(%)**	113 (9.7)	44 (10.9)	69 (9.1)	0.321

*GDS, geriatric depression scale (score range: 0-15); M, mean; MMSE, mini-mental state examination (score range: 0–30); SD, standard deviation*.

* *Based on the total score form the Lubben Social Network Scale (LSNS-6, scoring range: 0-30), which defines social isolation as a score below 12 and social integration as a score equal 12 or higher*.

### Effects of Social Isolation on Incident Dementia

Table 2 presents the results of the competing risk analysis. Social isolation was not significantly associated with incident dementia, neither in the unadjusted (sHR: 1.24, *p* = 0.26) nor in the adjusted model (sHR: 1.07, *p* = 0.80). In separate models for women and men, a significant association between social isolation and incident dementia was found in the unadjusted model for women (sHR = 1.46, *p* = 0.08), but not for men (sHR = 0.68, *p* = 0.38; see Table 3). After adjusting for possible confounders, no significant results were found for both women (sHR: 1.39, *p* = 0.27) and men (sHR: 0.71, *p* = 0.48). In separate models for women and men, no significant results were found for both women and men in the unadjusted (women: sHR = 1.46, *p* = 0.08; men: sHR = 0.68, *p* = 0.38; see Table 3) and the adjusted model (women: sHR: 1.39, *p* = 0.27; men: sHR: 0.71, *p* = 0.48). Results of the sensitivity analysis did not differ (see Supplementary Tables S1, S2).

**Table 2:** Univariate and multivariate Fine and Gray (competing risk) regression model for the impact of social isolation on incident dementia.

	**Model I**		**Model II**	
	***sHR***	***p***	***sHR***	***p***
Social isolation (ref. socially integrated individuals)	1.24	0.260	1.07	0.800
Age^*^			1.04	0.320
Male sex (ref. female sex)			*0.55*	*0.057*
High education (ref. middle, low)			*1.64*	*0.003*
Married (ref. not married)			1.40	0.288
Living alone (ref. shared housing)			1.08	0.788
Cognitive function (MMSE)^*^			*0.71*	*<0.001*
Depressive symptoms^*^			1.03	0.596
IADL^*^			1.00	0.965
Physical activities^*^			1.00	0.943
Cognitive activities^*^			1.00	0.905
Vision impairment (ref. no impairment)			0.70	0.228
Hearing impairment (ref. no impairment)			0.91	0.712
Mobility impairment (ref. no impairment)			1.03	0.920
Hypertension (ref. no history of hypertension)			0.80	0.463
Diabetes (ref. no history of diabetes)			*0.50*	*0.020*
Stroke (ref. no history if stroke)			1.21	0.648
*n*		1.161		843

* *Continuous scores; IADL, instrumental activities of daily living; MMSE, mini-mental state examination; sHR, subdistribution hazard ratio. Statistics in italicized type indicate significant results*.

**Table 3:** Univariate and multivariate Fine and Gray (competing risk) regression model for the impact of social isolation on incident dementia by gender.

	**Women**				**Men**			
	**Model I**		**Model II**		**Model I**		**Model II**	
	***sHR***	***p***	***sHR***	***p***	***sHR***	***p***	***sHR***	***p***
Social isolation (ref. socially integrated individuals)	*1.46*	*0.082*	1.39	0.274	0.68	0.375	0.71	0.479
Age^*^			1.03	0.487			1.08	0.279
High education (ref. middle, low)			*1.93*	*0.003*			1.42	0.170
Married (ref. not married)			1.03	0.948			*5.52*	*<0.001*
Living alone (ref. shared housing)			0.95	0.878			*5.43*	*0.003*
Cognitive function (MMSE)^*^			*0.70*	*<0.001*			*0.69*	*0.011*
Depressive symptoms^*^			1.04	0.497			0.89	0.264
IADL^*^			1.03	0.761			0.98	0.891
Physical activities^*^			0.94	0.256			1.06	0.444
Cognitive activities^*^			1.04	0.228			*0.85*	*0.011*
Vision impairment (ref. no impairment)			0.62	0.186			1.04	0.947
Hearing impairment (ref. no impairment)			0.83	0.547			1.18	0.731
Mobility impairment (ref. no impairment)			1.39	0.396			0.49	0.311
Hypertension (ref. no history of hypertension)			0.96	0.905			0.50	0.191
Diabetes (ref. no history of diabetes)			*0.41*	*0.022*			0.94	0.896
Stroke (ref. no history if stroke)			0.71	0.577			*3.06*	*0.073*
*n*		778		544		383		299

* *Continuous scores; IADL, Instrumental activities of daily living; MMSE, Mini-Mental state examination; sHR, subdistribution hazard ratio. Statistics in italicized type indicate significant results*.

## Discussion

We aimed to longitudinally investigate effects of social isolation on incident dementia in a large sample of oldest-old individuals taking into account the competing risk of mortality. Social isolation was highly prevalent in our sample (34.7%). Moreover, mortality was higher in socially isolated individuals compared to socially integrated individuals. We did not find an association between social isolation and incident dementia in the oldest-old, when taking mortality into account. Moreover, there was no association between social isolation and incident dementia in men or women.

There are a few studies that have also examined the association between social isolation and dementia or cognitive functioning in the oldest old (23, 36, 37). A study also based on AgeCoDe/AgeQualiDe data examined oldest-old, healthy individuals over a period of 4.7 years with regards to social isolation and cognitive function. It was shown that smaller social networks, measured with the LSNS-6, were associated with lower cognitive function (23). In addition, Hajek et al. (37) studied oldest-old individuals based on AgeCoDe/AgeQualiDe data with the LSNS-6 over a 2-year period and found that a social network size was associated with functional deterioration in men. The different findings in comparison to our study could be explained by varying methodological approaches. For example, in both previous studies, continuous outcomes were used. For this study, however, a defined clinical disease (dementia) was used as outcome. In addition, in contrast to Röhr et al. (23) and Hajek et al. (37), our analyses was adjusted for mortality risk by performing a competing risk analyses.

Other studies rather investigated social isolation in younger old age groups in relation to cognitive function instead of incident dementia (38–40). The results contradict the findings of our study. For example, Crooks et al. (38) conducted a longitudinal study with older women (78 and older) over 4 years. They showed that a larger social network had a protective effect on cognitive function in older women (38). Findings from Evans et al. (40) suggested that being isolated in late life is detrimental to cognitive function. They conducted a longitudinal study over 2 years with individuals aged over 65 years (40).

A study by Rodriguez et al. (41) considered individuals aged 75 years or older over 9 years. The results showed, in contrast to our findings, that having a restricted social network, assessed using the Wenger's Practitioner Assessment of Network Type (PANT), doubled the risk for developing dementia (41).

In a study over a 10-years period with dementia-free individuals who were 50 years old or older, Rafnsson et al. (42) found no association between social isolation and the development of dementia, maybe due to the relatively young age of the participants. Social isolation was operationalized using an index which included the extent of contact with the individual's social network and involvement in social organizations (42).

It is wellknown that women are at increased risk of developing dementia (43, 44). The gender difference can be explained in that women live longer than men in general. However, as individuals get older, the risk of developing dementia also increases (45). In addition, hormonal differences between men and women may be another reason why women are more likely to develop dementia. Moreover, differences in brain networks as well as in social, economic and cultural norms as well as relationships may contribute to differential dementia risk between men and women (45). There are also differences in the social networks of men and women. Because women live longer than men they are more likely to live without a spouse in old age (46). In addition, they have larger close, supportive networks as men (46). Schwartz et al. (46) found that the social networks of older European women grew over a time period of 4 years. Women have been shown to have greater relative increase in closer social relationships than men. This was despite the fact that there were no gender differences with the loss of number of confidants. Thus, women seem to tend to create new closer relationships, or add peripheral contacts to closer contacts (46). In a sample of older Koreans, Lee et al. (47) found that the cognitive function of women was influenced by social activity and the number of individuals they considered friends. Although these results might suggest that the influence of social network varies by gender, we did not find a significant association between social isolation and incident dementia in the unadjusted model as well as after adjusting for possible confounder for both oldest-old women and men.

Overall, most studies confirmed an association between social isolation and incident dementia or cognitive function. There may be several reasons why our results were not in line with previous studies. First, our follow-up period was rather short. A longer observation period may have provided differential insights. Second, our results may be explained by selective mortality (13, 48), i.e., individuals with a history of social isolation may not have reached oldest-old age in the first place. Thus, the individuals under investigation in this study may be rather resilient and have had a lifestyle that makes successful aging more likely. The four areas of preventing illness and disability; high cognitive, mental, and physical functioning; active participation in life; and good psychological adjustment in later life have been found to be important for successful aging (49). There is also evidence that physical activities (50), education, work life, leisure activities, stress, and diet are important factors for successful aging and health in late life (51). The difference in findings could be also explained due to heterogeneity of the study samples. In addition, previous studies have often used continuous score for cognition rather than dementia as a binary outcome. Cognitive scores can be used to detect more subtle changes than using a binary diagnostic outcome that represents solid clinical levels of impairment, such as the one used in our work.

Social isolation may not have been a phenomenon over the life course for many oldest-old individuals, but may rather be a correlate of the increasing age and survival, which is associated with decreasing social networks, for example, because of widowhood, the death of siblings and friends (52). With other words, social isolation may be more detrimental to cognitive function if it occurs during earlier late life and if it occurs over rather longer periods. This supports the general relevance for studying modifiable risk factors for dementia with regards to different age spans from a life course perspective in order to determine best practices of dementia risk reduction (53). Therefore, it would be useful if individuals were observed over a long period of time over the whole life-course, ideally starting in early life and continuing into oldest-old age to answer at what stage of life social isolation is a risk factor (e.g., adolescence); how long an individual must continuously live in social isolation before it becomes a risk factor; and whether the risk can be reversed when the individual is no longer affected by social isolation after a certain period of time. An example of how risk for dementia varies depending on age are hypertension and obesity. For example, studies found that systolic blood pressure levels conveying the lowest dementia risk differ between age groups and have rather *U*-shaped relation with dementia risk (54). Similar findings have been reported for obesity in relation to dementia risk (55). We suggest there may be a similar relationship with regards to social isolation as a risk factor for dementia.

In this context, it would furthermore be important to investigate whether feelings of loneliness have a different effect on the development of dementia in the oldest-old. In general, a *U*-shaped relationship between age and loneliness can be observed (56, 57). Social interactions that provide a sense of satisfaction and sociability have been shown to be a protective factor for dementia over 15 years (58). The presence of a confidant also has a protective effect (58). The likelihood of developing dementia symptoms is twice as high in individuals who feel lonely (58). This effect is also seen the other way around: people with AD are more likely to be lonely (58). Therefore, loneliness may be the better indicator to investigate research questions about cognitive health in the oldest-old.

## Strengths and Limitations

Strengths of the study include the large sample of oldest-old individuals who provide longitudinal data over an observation period of over 4 years. Second, comprehensive structured clinical interviews, and consensus conferences with clinical experts were conducted to diagnose incident dementia. Using competing risk analysis allowed us to adjust for cumulative risk of mortality (59). This is an important aspect in survival analyses, particularly in oldest-old individuals, and may yield more accurate risk associations with dementia. In our study, more than one third of the participants died during the study period (*n* = 418; 36.0%), with higher mortality in socially isolated oldest-old individuals. This finding highlights the methodological necessity to conduct competing risk analysis in survival analysis, in oldest-old populations, and may yield more accurate results.

The study has also limitations. First, the generalizability of the results might be limited because of a moderate response rate of individuals to the study and a substantial number of participants who could not be located or refused participation in follow-up assessments, which may bias our analytical sample toward healthier participants. Therefore, the results may represent an underestimation of the impact of social isolation and incident dementia. Second, our measure for social isolation, the LSNS-6, does not capture qualitative aspects of social isolation. Therefore, it cannot be clarified whether other aspects of a social network, for example, perceived social support or feelings of loneliness, have an effect on the development of dementia. It is known that there are individuals who prefer to be alone and may not be affected by having only few other people around them. They may not feel lonely, despite having a few social contacts. Other individuals may feel lonely even among a large social network. Without assessing qualitative aspects of a social network, conclusions remain limited. Third, the study group is dynamic in terms of social network characteristics. Therefore, we conducted a sensitivity analysis with social isolation as a time-varying variable. The results did not differ from the competing risk analysis.

Though we used a standardized screening measures to assess the risk of social isolation, it is difficult to compare the results with other studies because social isolation is operationalized differently in various studies.

Moreover, it was not possible for us to control the analysis for other potential variables that increase the risk of dementia.

## Conclusion

In contrast to the findings of previous studies, we did not find an association between social isolation and incident dementia specifically in the oldest-old. Consequently, social isolation may not be a risk factor for dementia in the oldest-old. This finding could be explained by selective mortality on the one hand and by a rather short study period on the other hand. The results highlight the importance of studying modifiable risk factors for dementia concerning age, as the impact of a risk factor may vary depending on life stages, e.g., midlife, early late life, or oldest-old age. This has important implications for precise prevention of cognitive decline and dementias.

## Author's Note

The results of the manuscript were presented at the 56th Annual Meeting of the DGSMP on 23/09/2021. The abstract has already been published in an abstract collection in the journal “Das Gesundheitswesen” (Georg Thieme Verlag, issue 8/9 2021). It can be downloaded under the following link: https://www.thieme-connect.de/products/ejournals/conferencepdf/079613/10.1055/s-00000022.pdf

## Data Availability Statement

The data analyzed in this study is subject to the following licenses restrictions: the dataset is available for research purposes upon reasonable request to the Data Handling Center of the Agecode/Agequalide Study. Requests to access these datasets should be directed to BW, Wiese.Birgitt@MH-Hannover.de.

## Ethics Statement

The studies involving human participants were reviewed and approved by Ethics Committee of the Medical Faculty of the University of Leipzig (Germany). The patients/participants provided their written informed consent to participate in this study.

## Author Contributions

SR, MS, MW, and SR-H: study concept and design. SR, KH, MP, AF, H-HK, BW, SW, JW, HB, DW, MS, MW, and SR-H: acquisition of data. SR, JG, AP, and SR-H: analysis and interpretation of data. JG and SR: drafting the manuscript. All authors: critical revision of the manuscript for important intellectual content. All authors contributed to the article and approved the submitted version.

## Funding

This work was supported by part of the German Research Network on Dementia (KND), the German Research Network on Degenerative Dementia (KNDD; German Study on Ageing, Cognition and Dementia in Primary Care Patients; AgeCoDe), and the Health Service Research Initiative [Study on needs, health service use, costs and health-related quality of life in a large sample of oldest-old primary care patients (85 +; AgeQualiDe)] and was funded by the German Federal Ministry of Education and Research (Grants KND: 01GI0102, 01GI0420, 01GI0422, 01GI0423, 01GI0429, 01GI0431, 01GI0433, and 01GI0434; Grants KNDD: 01GI0710, 01GI0711, 01GI0712, 01GI0713, 01GI0714, 01GI0715, and 01GI0716; Grants Health Service Research Initiative: 01GY1322A, 01GY1322B, 01GY1322C, 01GY1322D, 01GY1322E, 01GY1322F, and 01GY1322G). The publication was also supported by the study “Healthy Aging: Gender specific trajectories into latest life” (AgeDiferent.De) that was funded by the German Federal Ministry of Education and Research (Grants 01GL1714A, 01GL1714B, 01GL1714C, and 01GL1714D). We get DEAL for APC to the institution (University of Leipzig).

## Conflict of Interest

The authors declare that the research was conducted in the absence of any commercial or financial relationships that could be construed as a potential conflict of interest.

## Publisher's Note

All claims expressed in this article are solely those of the authors and do not necessarily represent those of their affiliated organizations, or those of the publisher, the editors and the reviewers. Any product that may be evaluated in this article, or claim that may be made by its manufacturer, is not guaranteed or endorsed by the publisher.
