# Supplementary material for: Social Isolation and Incident Dementia in the Oldest-Old—A Competing Risk Analysis
Source: Front Psychiatry. 2022 Jun 10;13:834438. doi: 10.3389/fpsyt.2022.834438 (PMC9226337; doi:10.3389/fpsyt.2022.834438)
Supplement: Supplementary file 1 [file Table_1.docx]

# Supplementary Material

*Table S1: Univariate and multivariate Fine and Gray (competing risk) regression model for the impact of social isolation on incident dementia.*

|  | Model S.I | | Model S.II | |
| --- | --- | --- | --- | --- |
|  | *sHR* | *p* | *sHR* | *p* |
| Social Isolation (ref. socially integrated individuals)° | 1.20 | 0.320 | 1.05 | 0.826 |
| Age^*^ |  |  | 1.04 | 0.326 |
| Male sex (ref. female sex) |  |  | *0.60* | *0.088* |
| High education (ref. middle, low) |  |  | *1.62* | *0.002* |
| Married (ref. not married) |  |  | 1.24 | 0.471 |
| Living alone (ref. shared housing) |  |  | 1.04 | 0.878 |
| Cognitive function (MMSE)^*^ |  |  | *0.74* | *< .001* |
| Depressive symptoms^*^ |  |  | 1.03 | 0.560 |
| IADL^*^ |  |  | 0.98 | 0.834 |
| Physical activities^*^ |  |  | 1.01 | 0.820 |
| Cognitive activities^*^ |  |  | 1.00 | 0.986 |
| Vision impairment (ref. no impairment) |  |  | 0.73 | 0.242 |
| Hearing impairment (ref. no impairment) |  |  | 0.98 | 0.921 |
| Mobility impairment (ref. no impairment) |  |  | 1.09 | 0.750 |
| Hypertension (ref. no history of hypertension) |  |  | 0.80 | 0.415 |
| Diabetes (ref. no history of diabetes) |  |  | *0.52* | *0.020* |
| Stroke (ref. no history if stroke) |  |  | 1.17 | 0.664 |
| *n* |  | 1.161 |  | 843 |
| *Abbreviations*.° Social isolation as time-varying variable; ^*^Continuous scores; IADL, Instrumental Activities of Daily Living; MMSE, Mini-Mental State Examination; sHR, subdistribution hazard ratio. | | | | |

*Table S2: Univariate and multivariate Fine and Gray (competing risk) regression model for the impact of social isolation on incident dementia by gender.*

|  | Women | | | | Men | | | |
| --- | --- | --- | --- | --- | --- | --- | --- | --- |
|  | Model I | | Model II | | Model I | | Model II | |
|  | *sHR* | *p* | *sHR* | *p* | *sHR* | *p* | *sHR* | *p* |
| Social Isolation (ref. socially integrated individuals)° | *1.37* | *0.125* | 1.25 | 0.393 | 0.70 | 0.408 | 0.85 | 0.723 |
| Age^*^ |  |  | 1.04 | 0.393 |  |  | 1.07 | 0.278 |
| High education (ref. middle, low) |  |  | *1.83* | *0.002* |  |  | 1.46 | 0.117 |
| Married (ref. not married) |  |  | 0.93 | 0.882 |  |  | *4.69* | *< .001* |
| Living alone (ref. shared housing) |  |  | 0.90 | 0.729 |  |  | *4.68* | *0.002* |
| Cognitive function (MMSE)^*^ |  |  | *0.73* | *< .001* |  |  | *0.74* | *0.006* |
| Depressive symptoms^*^ |  |  | 1.04 | 0.503 |  |  | 0.92 | 0.373 |
| IADL^*^ |  |  | 1.01 | 0.932 |  |  | 1.03 | 0.849 |
| Physical activities^*^ |  |  | 0.96 | 0.390 |  |  | 1.06 | 0.404 |
| Cognitive activities^*^ |  |  | 1.04 | 0.208 |  |  | *0.88* | *0.014* |
| Vision impairment (ref. no impairment) |  |  | 0.67 | 0.203 |  |  | 1.01 | 0.989 |
| Hearing impairment (ref. no impairment) |  |  | 0.89 | 0.649 |  |  | 1.22 | 0.625 |
| Mobility impairment (ref. no impairment) |  |  | 1.44 | 0.279 |  |  | 0.53 | 0.306 |
| Hypertension (ref. no history of hypertension) |  |  | 0.94 | 0.866 |  |  | 0.54 | 0.221 |
| Diabetes (ref. no history of diabetes) |  |  | *0.43* | *0.014* |  |  | 1.04 | 0.938 |
| Stroke (ref. no history if stroke) |  |  | 0.80 | 0.684 |  |  | *2.44* | *0.084* |
| *n* |  | 778 |  | 544 |  | 383 |  | 299 |
| *Abbreviations*. ° Social isolation as time-varying variable; ^*^Continuous scores; IADL, Instrumental Activities of Daily Living; MMSE, Mini-Mental State Examination; sHR, subdistribution hazard ratio. | | | | | | | | |
